# Continental-scale patterns in diel flight timing of high-altitude migratory insects

**DOI:** 10.1098/rstb.2023.0116

**Published:** 2024-06-24

**Authors:** Birgen Haest, Felix Liechti, Will L. Hawkes, Jason Chapman, Susanne Åkesson, Judy Shamoun-Baranes, Anna P. Nesterova, Vincent Comor, Damiano Preatoni, Silke Bauer

**Affiliations:** ^1^ Swiss Ornithological Institute, Seerose 1, Sempach, 6204, Switzerland; ^2^ Swiss Birdradar Solution AG, Technoparkstrasse 2, 8406, Winterthur, Switzerland; ^3^ Centre for Ecology and Conservation and Environment and Sustainability Institute, University of Exeter, Cornwall Campus, Penryn, TR10 9FE, UK; ^4^ Department of Entomology, Nanjing Agricultural University, Nanjing, 210095, People's Republic of China; ^5^ Department of Biology, Centre for Animal Movement Research, Lund University, Ecology Building, 22362 Lund, Sweden; ^6^ Theoretical and Computational Ecology, IBED, University of Amsterdam, P.O. Box 94240, Amsterdam, GE 1090, The Netherlands; ^7^ Oréade-Brèche, 70, rue de l'Eglise, 67130 Schirmeck, France; ^8^ Independent researcher, Les Pennes-Mirabeau, 13170, France; ^9^ Department of Theoretical and Applied Sciences, University of Insubria, Via J.-H. Dunant 3, Varese, 21100 Italy

**Keywords:** insect activity, diel flight activity, vertical-looking radar, insect migration, flight periodicity, circadian rhythm

## Abstract

Many insects depend on high-altitude, migratory movements during part of their life cycle. The daily timing of these migratory movements is not random, e.g. many insect species show peak migratory flight activity at dawn, noon or dusk. These insects provide essential ecosystem services such as pollination but also contribute to crop damage. Quantifying the diel timing of their migratory flight and its geographical and seasonal variation, are hence key towards effective conservation and pest management. Vertical-looking radars provide continuous and automated measurements of insect migration, but large-scale application has not been possible because of limited availability of suitable devices. Here, we quantify patterns in diel flight periodicity of migratory insects between 50 and 500 m above ground level during March-October 2021 using a network of 17 vertical-looking radars across Europe. Independent of the overall daily migratory movements and location, peak migratory movements occur around noon, during crepuscular evening and occasionally the morning. Relative daily proportions of insect migration intensity and traffic during the diel phases of crepuscular-morning, day, crepuscular-evening and night remain largely equal throughout May-September and across Europe. These findings highlight, extend, and generalize previous regional-scale findings on diel migratory insect movement patterns to the whole of temperate Europe.

This article is part of the theme issue ‘Towards a toolkit for global insect biodiversity monitoring’.

## 1. Introduction

During times of year that environmental conditions permit, trillions of insects take to skies for high-altitude migratory movements [1–3]. Many of these insects provide essential ecosystem services. Others are pests that threaten biodiversity, cause substantial economic damage, or pose a risk to human health [4–7]. Throughout these aerial displacements, many are also feeding or preyed upon, and thus contribute to trophic interactions with resident and other migratory animals [1,4,6,8]. Even though these migratory movements are widespread, occur over large distances, involve innumerous individuals, and have important implications for biodiversity conservation and pest management, their scale, magnitude, extent and daily timing have remained largely unexplored. Studies have mostly remained local and relatively short-term (e.g. some days or weeks) because traditional trapping methods quickly become too costly and impractical for long-term, temporally detailed and geographically spread data acquisition [9,10]. However, the recent reports on, often dramatic, declines in insect populations [11–14] and their suspected knock-on effects further up the food chain [15] urge for a better understanding of migratory insect movements through long-term and large-scale monitoring [6,16].

Migratory flights of insects often begin with ascending several tens to hundreds of meters out of their ‘flight boundary layer’, i.e. the relatively narrow layer near the ground where their flight speed exceeds wind speed, to profit from favourable winds higher up in the air [7,17–20]. These flights are typically periodic, i.e. most insect species only fly during part of the 24 h daily cycle [21]. Two basic options exist for the time-of-day of migratory flight: diurnal migrants profit from higher air temperatures and better illumination (which presumably helps to orientate), and nocturnal migrants benefit from stable vertical air layers, i.e. with little convective up- or down-draughts, allowing better control of their altitude to take advantage of warm and high-speed air currents in the desired direction [7,22,23]. Diurnal migration generally commences around mid-morning, as atmospheric convection develops, and ends sometime in the late afternoon. Nocturnal migrants generally take off at dusk and some fly throughout the night. Additionally, crepuscular-only species take off during the dawn twilight period and after a short period of flight, land again around sunrise [17,23,24]. These three diel periods of migratory activity typically involve distinct taxa with little overlap [21,23].

Initial insights into the circadian flight activity of migratory insects stem from ground or low-altitude trapping, with some programmes such as the Rothamsted suction trap networks in the UK and USA using standardized protocols that run over longer periods of time. However, such trapping only provides flight activity data for the first few (typically around 15) meters above the ground [10,21,25]. Sometimes, ground trapping has been extended to higher altitudes by putting trapping devices on tall towers, aircrafts, balloons, or kites [26,27]. The higher associated costs of such aerial trapping, however, restricts their use to short periods [1,9,10]. For long-term, automated monitoring of aerial insect migration over a wide range of altitudes, only vertical-looking radars and lidars are available [7,28–30]. These techniques provide information on the size (or mass), wing beat frequency, speed, direction of flight and flight altitude of insects [31,32]. While insect monitoring with lidar remains largely experimental, with studies performed over limited measurement periods only, vertical-looking radars have become the tool of choice, and most of what we know about aerial migratory insect movements has been derived from the use of these radars [9,16,23,33]. While some research programmes have run for several years, the number of radars involved has been limited to a few devices only. Consequently, the geographical coverage of simultaneous measurement and, thus, results obtained, remain limited and cannot easily be scaled up [34] (but see [3] for a study covering southern UK with three vertical-looking radars, the largest spatial cover studied so far).

Here we present the daily variation in migratory insect movements between 50 and 500 m above ground level (AGL) across Europe from March to October 2021, as detected by 17 Birdscan MR1 vertical-looking radars. Our key objectives were to identify and quantify the circadian patterns in aerial migratory insect movements at various locations in western, central, and northern Europe and to investigate the variation in these circadian patterns across the year and locations. To do so, we quantified and compared hourly insect migration traffic rates, as well as daily average migration intensity and traffic during four diel phases, i.e. day, crepuscular evening, night and crepuscular morning, with crepuscular times being defined as the times when the sun's geometric centre is between 12° and 0° below the horizon. This paper is, to our knowledge, the first study ever to simultaneously measure migratory insect movements at various points across the European continent. Because in this study, we specifically aimed to investigate spatiotemporal variation in circadian patterns of migratory insect movements and not changes in absolute numbers of insects across the season, we focused our analyses on the variation in relative daily proportions of insect migration traffic and rates. We also compared patterns in median flight altitude and radar cross sections (RCS; as a proxy of insect mass) during each of the diel phases to identify potential differences in the migratory insect community composition and their flight altitude behaviour across the locations and during each of the diel phases. Aspects other than the diel cycle (e.g. seasonality and environmental influences) will be the subject of further publications.

## 2. Methods

### (a) Radar measurements

We used a network of BirdScan MR1 (software v1.6.0.12) 25 kW X-band (9.4 GHz, 3.2 cm wavelength) pulse radars with an optionally rotating Horn antenna (nominal beam width at −3 dB is approx. 17.5°) [35,36] to measure aerial insect movements. The Birdscan MR1 radar can be operated in different modes (e.g. with or without rotation and using different pulse lengths). Here, we only retained data from times in which the radars were operating in short-pulse mode (i.e. pulse length 65 ns, pulse rate frequency 1800 Hz, range resolution about 7.5 m), and with a nutating antenna (rotation frequency is about 0.8 Hz with an angle offset from the vertical axis of 2°). Using a dataset of annotated radar echo samples [37], the probability to be an insect, one of several subgroups of birds or a non-biological object is calculated for each detected object [35,38]. To improve insect identification specifically, the classifier includes polarization shape features [39]. We retained only those objects for which the probability to be an insect was higher than 0.4 and higher than for any of the other classes.

**Figure 1.**
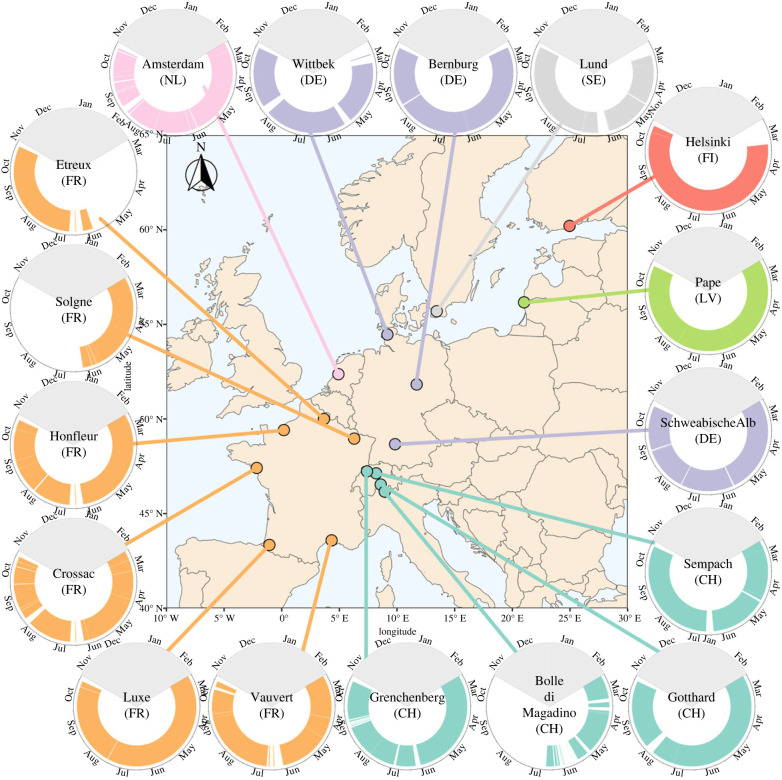
Radar measurement locations (map) and times (circle plots). Coloured bars in the circle plots indicate the times during which measurements were made at that location in 2021. The names in the centre of the circle plots are the municipalities of the radar locations. The locations are colour-coded per country. The grey part of the circle plot indicates the time of year we did not assess.

The network comprised 17 radars that were located along a southwest to northeast axis from north of the Pyrenees in southwestern France to Helsinki in Finland (figure 1; electronic supplementary material, table S1). Latitudinally, the radar locations ranged from approximately 43° to 60° N and longitudinally from about 2° W to 24° E. Amsterdam in The Netherlands was the site with the lowest elevation at 1 m and Gotthard in Switzerland the highest at 1544 m above sea surface level. We aimed to measure continuously at all locations from 1 March to 31 October 2021 but for some locations, measurements were interrupted owing to logistic or technical reasons (figure 1; electronic supplementary material, figure S1). Across the 17 sites, we measured during 3784 days in total.

### (b) Quantification of diel migratory insect movements

For all radars, the insect echo counts between 50 and 500 m AGL (i.e. above the radar) were first converted into non-directional insect traffic rates (i.e. the number of insects per km and hour) for 1 h time bins across the whole measurement period using the ‘*birdscanR*’′ R package [40]. If the effective monitoring time for a certain 1 h time bin fell below 12 min (i.e. 20%), e.g. because of technical shut-down, we set the insect traffic rates in that 1 h time bin to NA. All identified rain events were treated as times of zero insect traffic.

The length of days, twilights, and nights varied substantially across our measurement period and geographical locations, so, beyond the purpose of visualization, comparing the hourly insect traffic rates provides little insights into the spatiotemporal variation in diel migratory insect flight activity. We therefore calculated two additional measures to express migratory insect traffic during each of four diel phases: (i) the mean insect traffic rate, i.e. the average number of insects per km and hour during the respective daily diel phase, and (ii) the total insect traffic, i.e. the total number of insects per km passing throughout the full one-day period of the diel phase. We defined the four diel phases as follows: ‘crepuscular morning’ is the interval between nautical dawn and sunrise (i.e. geometric centre of the sun between 12° and 0° below the horizon in the morning), ‘day’ is the period between sunrise and sunset (i.e. geometric centre of the sun between 0° below and above the horizon), ‘crepuscular evening’ is the interval between sunset and nautical dusk (i.e. geometric centre of the sun between 0° and 12° below the horizon in the evening), and ‘night’ is between nautical dusk and nautical dawn (i.e. geometric centre of the sun between 12° below the horizon in the evening and in the morning). At the three most northern sites, i.e. Lund (Sweden), Pape (Latvia) and Helsinki (Finland), there was no ‘night’ during part of the summer months, so we assigned insects that flew during the first half of the full twilight time to crepuscular evening and those that flew during the second half to crepuscular morning.

As we were specifically interested in the spatiotemporal variation in diel migratory insect activity without the confounding effects of seasonality that change the absolute numbers of daily total migratory insects, we converted both the mean daily insect traffic rate and the total daily insect traffic during each of the diel phases to their relative daily proportions. We did this by dividing the mean insect traffic rate during a diel phase by the sum of the mean insect traffic rates during all diel phases of that day, and similarly, by dividing the total insect traffic during a diel phase by the total insect traffic across all diel phases during that day. Both measures thus can vary between 0 and 1 and sum to 1 across all diel phases per day. Throughout the manuscript, we refer to these two measures as the proportional migration intensity and traffic, respectively. Using daily proportions of mean insect traffic rates allows comparing how migration intensity during each of the diel phases varies across time and space in comparison to the migration intensity during other diel phases. Comparing these patterns with those of the daily proportions of overall traffic during each of the diel phases, then allows separation of the effect of variation in the relative migration intensity from those caused by the duration of the diel phase.

### (c) Analysis of patterns in diel migratory flight across space and time

To visually compare daily patterns in hourly insect traffic rates across the measurement period and locations, we created heatmaps of both the (absolute) hourly insect traffic rates and the relative daily proportions of the hourly insect traffic, i.e. a value between 0 and 1 indicating how much of the insect traffic of that day occurred during the respective hour. Heatmaps of the (absolute) hourly insect traffic rates provide good insights into the diel pattern during days of the year with high migratory insect traffic but obscure variation within days of relatively low insect traffic. Using relative daily proportions of the hourly insect traffic makes the daily patterns in hourly insect traffic rates visually apparent during days when overall insect traffic is rather low.

We used the proportional migration intensity and traffic to statistically test to which extent daily insect migration intensity and traffic during each of the diel phases varied between locations across Europe. For each diel phase, we first performed an ANOVA with beta distributions (because the data are proportional data, i.e. with values ranging from 0 and 1 [41,42]) to test for differences between the means of the proportional migration intensity and traffic of all locations. We excluded three sites (Etreux and Solgne in France, and Bolle Di Magadino in Switzerland) because their measurements did not cover the entire measurement period of March to October 2021, which might have resulted in misleading differences that are owing to seasonality instead of geographical location (electronic supplementary material, table S1). To avoid zeros and ones in the proportional data, we compressed the data using the formula *y*′ = (*y* × (*N* − 1) + 0.5)/*N*, where *N* is the sample size (i.e. number of days with values for the respective diel phase [43]. As the (omnibus) ANOVA analyses across all locations had a *p*-value < 0.05 for all diel phases (electronic supplementary material, table S2), we subsequently ran post-hoc ANOVA analyses to test which pairwise location-combinations (91 combinations for each of the four diel phases) of the proportional migration intensity and traffic differed. We adjusted the *p*-values of the post-hoc analyses using Holm correction [44] to account for the large number of tests. To explore the temporal variation in circadian insect migration traffic at each of the sites, we plotted monthly means of the proportional insect migration intensity and traffic during each of the diel phases.

### (d) Insect community composition and median altitudes during diel phases

Direct species identification is not possible with the Birdscan MR1. Certain features from the echo return signal are, however, related to individual characteristics such as mass and size. Here we use the low pass-filtered maximum radar cross section across all (beam rotation) polarizations (RCSmax from here on) as a proxy for insect mass. Although several factors influence the RCSmax, a higher RCSmax is expected to be a proxy for a higher mass [45]. We compared the average daily estimates of the RCSmax during each of the four diel phases across all locations to assess patterns in size composition.

We explored the spatio-temporal variation in altitudinal patterns by plotting median monthly altitudes during each of the diel phases for 13 of the study sites. In addition to the sites omitted owing to large gaps in the measurement period, we here also excluded Gotthard (Switzerland) owing to its different radar settings that could have influenced the average altitude estimates (figure 1; electronic supplementary material, table S1).

## 3. Results

### (a) Spatiotemporal patterns in the diel timing of aerial insect migration

**Figure 2.**
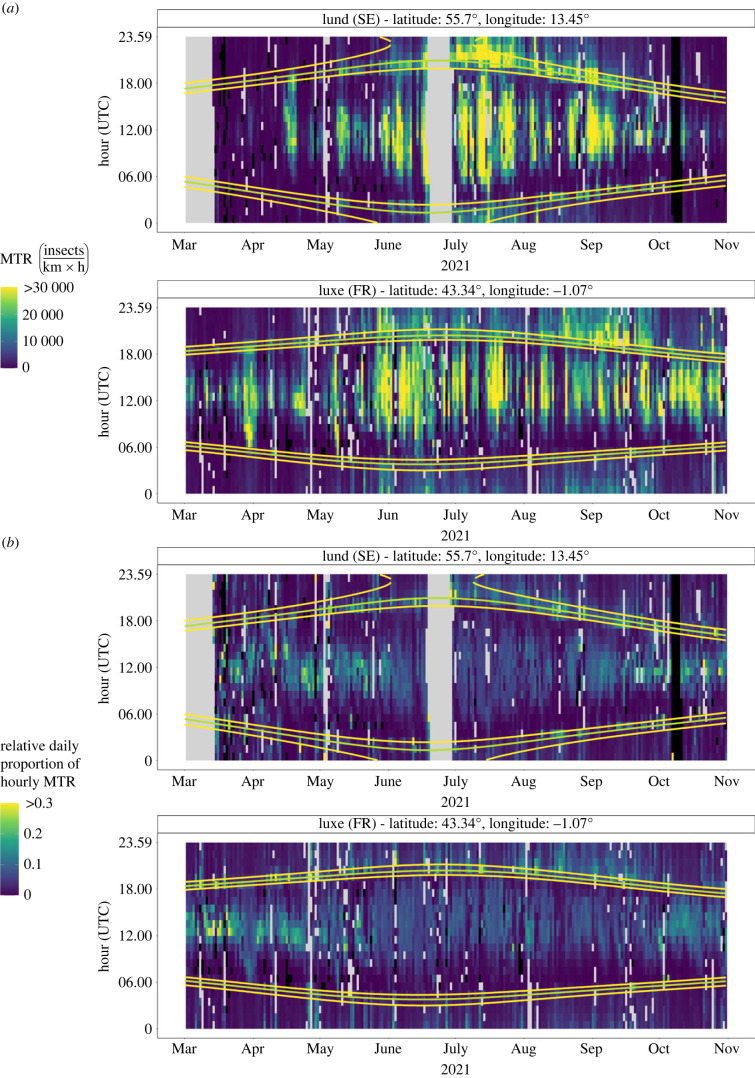
Hourly insect migration traffic rates (MTR) (*a*); (number of insects per hour and km) and daily proportional hourly insect traffic rates (*b*); a value between 0 and 1 indicating how much of the insect traffic of that day occurred during the respective hour) for one northern (Lund, Sweden) and one southern (Luxe-Sumberraute, France) sites. The yellow lines indicate sunrise/sunset and nautical dawn/dusk, and green lines times of civil dawn/dusk. For corresponding figures of all sites, see the electronic supplementary material, figures S2 and S3. Grey grid cells indicate times for which there was no data (NA values); black grid cells indicate times with a value of 0.

Daily patterns in hourly insect traffic rates (number of insect per km and hour; figure 2*a*; electronic supplementary material, figure S2) and daily proportions of hourly insect traffic rates (figure 2*b*; electronic supplementary material, figure S3), show a rather clear and consistent pattern in daily migratory insect movements throughout Europe. During times when many insects are migrating, i.e. most summer days and some of the spring and autumn days (especially May to September; figure 2*a*; electronic supplementary material, figure S2), migratory insect activity is relatively low just after sunrise, increases to a peak at around mid-day, slowly decreases towards dusk, peaks again during evening twilight, then again slowly decreases towards nautical sunrise, and on many days also shows another, albeit short and less intense peak at dawn. During times when much fewer insects are migrating (i.e. mainly March, April and October), migratory movements mainly occur during day-time and less during the night and twilight times (figure 2*b*; electronic supplementary material, figure S3).

**Figure 3.**
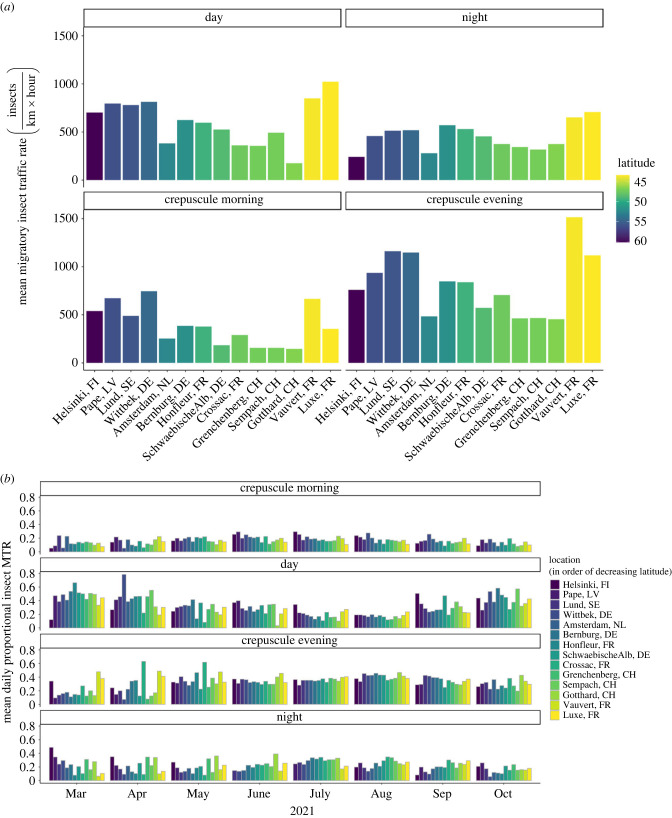
(*a*) Insect traffic rate (number of insects per km and hour) during four diel light phases, i.e. day, night, crepuscular morning and crepuscular evening, averaged across the whole measurement period at each of the locations; and (*b*) monthly means of proportional migration intensity, i.e. a value between 0 and 1 indicating the daily proportion of insect migration traffic during the respective diel phase, for each of the four diel light phases and the 14 locations for which measurements where made over the entire measurement period from March to October 2021.

When averaged across the whole measurement period (March to October), insect traffic rates were highest during crepuscular evening times, followed by day, and finally either night or crepuscular morning, depending on the measurement location (figure 3*a*). The monthly means of the proportional migration intensity (figure 3*b*), however, reveal that there is seasonal variation in this pattern. During the summer (May to August), the monthly means of proportional migration intensity remain rather stable and have similar distributions across the diel phases with traffic being most intense during the crepuscular evening times. At the beginning (March and April) and the end (October) of the measurement period, however, monthly mean proportional migration intensity was generally higher during the day than during the other diel phases. These patterns in monthly means of proportional migration intensity are largely consistent across all measurement locations. When adjusting for the duration of the diel phase, the monthly means of proportional migration traffic show that the largest proportion (25–75%) of insect movements occur during the day at most locations and during most months, followed by nightly (5–50%) and crepuscular evening (0–40%) movements, and finally by crepuscular morning movements with generally much lower monthly mean proportional migration traffic (0–15%) (electronic supplementary material, figure S4).

The pairwise effect sizes from the post-hoc ANOVA analyses, i.e. the differences in both the mean proportional migratory intensities and traffic, were largely normally distributed around 0 for all four diel phases, with significant differences having values ranging from 0.05 to 0.25 (absolute values, ignoring the direction of the effect; electronic supplementary material, tables S3 and S4, figures S5 and S7). About 30% of the pairwise differences were significant at the 0.05 level (i.e. 112 out of 364; electronic supplementary material, tables S3 and S4, figures S6 and S8). For the crepuscular morning phase, all pairwise differences were less than 0.1 for the proportional migration intensity and less than 0.04 for proportional migration traffic. The majority of the significant differences were because the proportional migration intensity and traffic during crepuscular morning in Amsterdam (The Netherlands) were higher than in other locations, while in Grenchenberg they were lower than in other locations. For the day values of proportional migration intensity and traffic, the maximum pairwise effect size was 0.16 and 0.24, respectively. The highest effect sizes were primarily for differences with the two sites at higher elevations (i.e. Gotthard and Grenchenberg in Switzerland), and with the western-most site in Crossac (France). Proportional migration intensity and traffic during the day were on average lower in these three sites compared to the other locations. For the crepuscular evening phase, the maximum difference in pairwise comparisons of the proportional migration intensity and traffic was 0.23 and 0.08, respectively. The two most southern sites of Luxe and Vauvert in France, and the most western site in Crossac (France) had higher proportions of migration intensity (and traffic, albeit to a lesser extent) during crepuscular evening. The second northern-most site of Pape (Latvia) had lower proportions of migration intensity and traffic during crepuscular evening. The differences in proportions of nightly migration intensity and traffic were below 0.15 and 0.23, respectively. The biggest differences in the nightly proportions mainly occurred because of the two sites at the highest elevation, i.e. Gotthard and Grenchenberg in Switzerland, having higher average nightly migration intensity and traffic than the other locations. Wittbek in northern Germany then again had lower proportions of nightly migration intensity and traffic.

### (b) Spatio-temporal patterns in insect community composition and median altitudes during the diel phases

**Figure 4.**
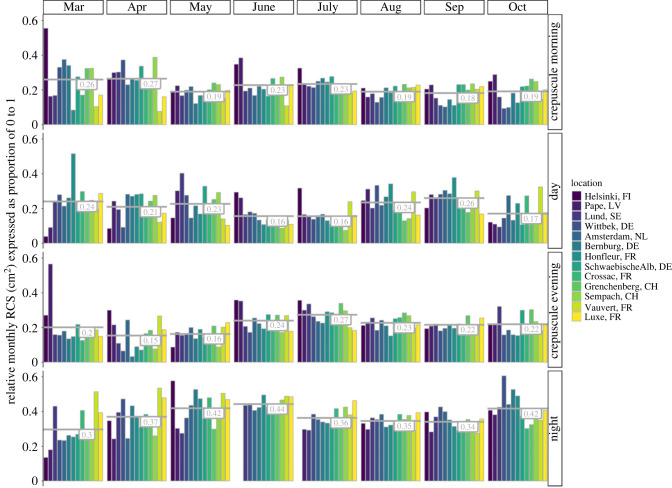
Relative mean monthly RCS, expressed as a proportion between 0 and 1, during each of the four diel light phases for 13 measurement sites across Europe. The grey numbers indicate the average relative mean monthly RCS proportion across all sites during that month. Proportions add up to 1 for each location and month.

**Figure 5.**
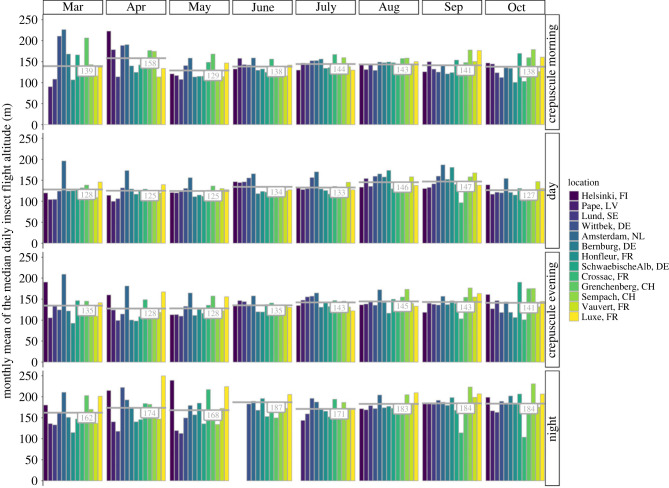
Monthly mean of the daily median migratory insect flight altitude (in metres above the radar) during each of the four diel light phases for 13 measurement sites across Europe. The grey numbers indicate the average flight altitude (in metre) across all sites during that month.

Mean monthly RCS values ranged from about 0.03 to 0.80 cm^2^ (electronic supplementary material, figure S9). In general, across locations and the eight-month measuring period, insects seemed to have considerably larger RCS values during the night than during any of the other diel phases (figure 4; electronic supplementary material, figure S9). The monthly mean flight altitude of insects varied from about 130 to 250 m above the radar (figure 5). On average, throughout the whole measurement period and for all locations, the mean monthly altitude of migratory insect movements was about 20 to 60 m higher above the radar during the night than during the other diel light phases. No obvious trends could be observed across the year or between sites.

## 4. Discussion

### (a) Spatiotemporal consistency in diel migratory insect flight timing across Europe

Overall, we found that the circadian pattern in migratory insect movements from March to October is largely consistent across Europe (figures 2 and 3). Throughout late spring to early autumn (i.e. roughly May to September), migratory insect movements are relatively low just after sunrise and then increase towards a mid-day peak. Thereafter, insect movement slowly decreases towards sunset, to again peak with a similar intensity during evening twilight. Throughout the night, it then again slowly decreases towards nautical sunrise, and on many days also shows another, albeit short-lasting and less intense peak during morning twilight (figure 2; electronic supplementary material, figures S2 and S3). These results are in line with and extend those from other regional scale radar studies in the southern UK [10,17,24], thus generalizing this daily pattern of migratory insect flight activity as a rather overarching pattern of migratory insect movements across most of Europe during times of the year when (temperature) conditions permit. We furthermore showed that the daily proportional distribution of both insect migration intensity and traffic between four diel phases, i.e. crepuscular morning, day, crepuscular evening, and night, is rather stable across this period of high migratory activity, i.e. from late spring to early autumn (figure 3*b*; electronic supplementary material, figures S4–S8). During colder times of the year (e.g. in our study mostly in March, April, and October), activity becomes largely limited to daytime and occurs especially around noon. Although the strong spatial consistency across a rather large geographical range suggests that these diel migratory insect activity patterns are probably also generalizable across years, our results from measurements across one year only do not allow assessing inter-annual fluctuations in these patterns.

Similar to what was shown for regional-scale insect movements in the southern UK [3], we show that also at the European scale most high-altitude migratory insect movements occur during the day, followed by the night, and then crepuscular times (with total movement during crepuscular evening being higher than during crepuscular morning; electronic supplementary material, figure S4). These high proportions of total insect air traffic, however, result partly from the duration of day and night being much longer than those of the crepuscular times, as insect migration traffic intensity (i.e. the number of insects per hour and km) is often higher during crepuscular evening than during day, but especially night times (figure 3). Crepuscular morning and night traffic intensity then again are very similar during most times and at most locations. *Post-hoc* ANOVA tests and the associated pairwise effect sizes statistically confirmed that indeed most of the distributions of proportional insect migration intensity and traffic during the diel phases show rather little difference across Europe (electronic supplementary material, tables S3 and S4, figures S5–S8). Some distributions did, however, significantly differ from others, indicating that there are factors other than light intensity also driving these daily distributions. Some of these differences probably result from latitude, elevation or other potential drivers such as habitat or landscape characteristics. Perhaps because the diel pattern of insect migratory flight activity is highly consistent across space when (temperature) conditions permit [19,20], few studies have specifically investigated the drivers of spatiotemporal variation in circadian distributions of migratory insect flight intensity and traffic. A recent study, however, did show differences in diel average hourly migratory insect traffic rates between several landscape types [46]. Future research endeavours are warranted to elucidate the drivers behind these occasional spatial (and temporal) differences.

The general consistency in insect migration intensity and traffic across space during most of late spring and summer powerfully illustrate how light intensity is the most important driver in the timing of overall daily insect movements across Europe [17,20,21]. The seasonal pattern of proportional migration intensity being highest for day during early spring and autumn, and for crepuscular evening during late spring and summer, then again indicates likely physiological limitations imposed by the seasonality of temperature [9,34,47]. Insects require temperatures above a certain threshold to become active, so temperature also determines their seasonal movement phenology and abundance (figure 2*a*; electronic supplementary material, figure S2). During late spring and summer, temperatures are typically high enough throughout the 24 h daily cycle to accommodate activity in species with different light intensity preferences. By contrast, activity-temperature thresholds are typically only reached during daytime in early spring and autumn, which restricts (lowers) activity and migratory movements of insect species with lower light intensity preferences (figures 2 and 3*b*; electronic supplementary material, figure S2). The daily illumination cycle, however, clearly seems to be the main factor shaping the relative distributions of insect migration intensity and traffic across the 24 h daily cycle.

Unfortunately, radars do not provide the taxonomic identities of the insects recorded. However, our results showed that the insects moving during night time were, on average, larger than the ones moving during crepuscular and day times (figure 4; electronic supplementary material, figure S9). These findings are in line with those from the regional-scale study over the southern UK [24], suggesting this is a general rule for insect movements across Europe (and the year). The altitudes at which insect aerial movements occur are thought to be determined by the concurrent aerial layering of both temperature and winds [19], although observed patterns remain diverse. Here, we showed that nightly movements, on average, take place at higher altitudes (above the ground) than during any of the other diel phases, independent of site elevation (figure 5). This finding extends similar observations from a radar study across Switzerland [46]. Given the size-dependent detection volume of vertical-looking radars [35], these patterns, however, need be interpreted with care.

### (b) Implications for and outlook on large-scale insect biomass monitoring using radar

In this study we used a network of 17 vertical-looking radars to quantify large-scale patterns in diel timing of migratory insect movements. Daily migration intensity and traffic were surprisingly consistent over large parts of the year, suggesting that measurements in one diel phase might be sufficient to estimate those numbers for other diel phases. Similarly, we found distributions to be largely similar across most of Europe, which would make interpolations between measurements locations possible. However, there were several exceptions to these patterns of similarity—the drivers of which warrant further study. Furthermore, if combined with automated ground insect identification systems (e.g. [48]), the currently coarse taxonomic resolution of radar observations could be refined. Such systems are increasingly being developed [49], and their integration with vertical-looking radar data could usher in a new era of insect radar aeroecology that allows for widespread monitoring of aerial insect biodiversity.

## Data Availability

The proportional migratory insect intensity and traffic data are available from the Zenodo repository: https://doi.org/10.5281/zenodo.10626690 [50]. Additional figures and tables are provided in the electronic supplementary material [51].
